# The reasons why patients abscond from public hospitals in southeastern Iran: a qualitative study

**DOI:** 10.1186/s13690-021-00634-z

**Published:** 2021-06-18

**Authors:** Mahnaz Moradpour, Mohammadreza Amiresmaili, Mahmood Nekoei-Moghadam, Tania Dehesh

**Affiliations:** 1 Health Services Management Research Center, Institute for Futures Studies in Health, Kerman University of Medical Sciences, Kerman, Iran; 2 Policy and Health Economics, Faculty of Management and Medical Information, Kerman University of Medical Sciences, Kerman, Iran; 3 Health in Disasters and Emergencies Research Center, Institute for Futures Studies in Health, Kerman University of Medical Sciences, Kerman, Iran; 4 Department of Biostatistics and Epidemiology, School of Public Health, Kerman University of Medical Sciences, Kerman, Iran

**Keywords:** Patient absconding, Public hospitals, Reasons, Iran

## Abstract

**Background:**

Patient absconding from hospital is one of the permanent issues the hospitals face, which poses many risks and challenges to the patient, hospital, and society. The present study aimed to identify the reasons for patient absconding behavior for public hospitals in southeastern Iran.

**Methods:**

The present study is a qualitative study which was conducted at three public hospitals in southeastern Iran using purposive sampling through semi-structured interviews with 63 informants involved in patient treatment process. Data were analyzed using Colaizzi content analysis (CCA) method.

**Results:**

Three main themes of economic, social factors, and factors related to the hospital covering 15 subthemes were identified to explain the reasons for patients absconding behavior.

**Conclusion:**

There are many reasons for reducing patients absconding from hospitals, and one of the main reasons is the patients’ economic and social problems. The absconding behavior can lead to harm and problems for patients, and some emotional and occupational consequences for the employees and nurses. Paying attention to this issue and considering some courses of action to prevent patient absconding might lead to a considerable promotion of public trust and eliminate many problems for hospitals.

## Background

Hospitals are one of the main elements of the healthcare system, and no health system can operate on its own without the participation and support of the hospitals [1]. However, hospitals face many problems and challenges, including patient absconding behavior. The patient absconding from hospital is one of the important health and security problems for health systems [2, 3].

Patient absconding behavior is defined as the patient leaving the hospital without informing staff and before completing the courses of treatment and paying their medical bills, which may put themselves and others at risk [4]. The patient absconding from hospital varies depending on the type of hospital (general and psychological). The most important reasons can be due to fatigue and hopelessness, intolerance of hospital, overdose of drugs, addiction, and poisoning, drinking alcoholic drinks, treatment failure, behavioral disorders, unemployment, and receiving unpleasant news [5]. However, the most important reason for patients to escape from general hospital is the inability to pay the medical costs, which has been mentioned in some studies [6, 7]. According to research, absconders are typically young, male, and have been diagnosed with schizophrenia [8–12].

This behavior can pose many risks, including longer recovering process or uncompleted treatment [13]. Also, it leads to an increase in hospital expenses and those of society. For instance, a study conducted in 2015 in the emergency department of an Iranian hospital indicated that the average medical expenses to be paid by each absconded patient was estimated at about 1.500.000 Rials,^1^ and the total loss made to the hospital was estimated at about 1.100.000.000 Rials [14].

On the other hand, the patients who abscond the emergency departments are often causing lots of problems for the staff, especially supervisors, hospital managers, security guards, since they have to spend a lot of time returning patients [15].

In many studies conducted around the world, the rate of patient absconding from hospital has been reported as 2.5 to 34%, most of which have been reported from the psychiatric units [11]. Also, in a study conducted in Iran, the rate of patient absconding from the emergency department of general hospitals was about 2.4% [14]. Considering the studies that have been conducted in other countries, the greatest average rates of patient absconding from hospitals were reported in America (8.92), England (6.28), India (5.58), Ireland (4.28), Iran (2.40) per 100 patients admitted to the hospitals (5).

In the southern and southeastern regions of Iran, often due to cultural issues, low income, and illegal residence of foreign nationals, hospitals in this area face the challenge of patient escape. Therefore, the reasons for patient escape from hospitals in these areas have not been addressed so far, and therefore health system policymakers do not understand the problem exactly.

Accordingly, the present study was conducted to investigate the reasons for patient absconding from three public hospitals in southeastern Iran.

## Method

### Definition of patient absconding

A patient who leaves the hospital unexpectedly before completing the medical examination and treatments without expressed permission. The patient absconding in this study means a person who leaves the hospital for 72 h without permission from the hospital staff and never returns.

### Study design

The present study is a qualitative study that was conducted through semi-structured interviews with informants. Recruitment was conducted by purposive sampling and snowball sampling. The informants were selected from people who were in connection with or involved in patient absconding behavior like the managers, physicians, nurses, patients carriers, security guards, and social workers from 3 public hospitals (i.e., purposive sampling), and some other participants were also recruited through recommendation by initial informants (i.e., snowball sampling) (Table 1). Accordingly, the people with a working experience of more than 5 years in the hospital who were willing to participate in the study were entered into the study.

**Table 1:** Themes and subthemes related to patients’ escape

Economic factors	Lack of insurance coverage
	Lack of proper and complete insurance coverage
	Inability to pay costs
	Illegal foreigner patients
Patients’ social and behavioral factors	Addiction
	Suicide and illegitimate pregnancies
	Free hospital services misconception
	Nonsupportive families
	Mental and psychological problems of patients
Hospital-related factors	The horizontal building structure of hospitals and insufficient supervision
	Not training and informing patients
	Absence of an established process for pursuing a patient escape
	Patients’ dissatisfaction with hospitals
	Difficulties in the discharge process of the patients

### Qualitative data collection

In this study, in-depth interviews were conducted with different groups of hospital staff to gain a full and detailed understanding of the interviewees’ experiences and beliefs about the reasons for patient absconding.

Interviews with absconders were not possible due to lack of access to these individuals (These people could help to better understand and study this phenomenon in more depth). In the general hospitals studied, most of the patients who escaped with the cooperation of their families and for various reasons were not motivated to return to the hospital and could not be reached.

Most of the interviewees were female (68.2%) and had more than 5 years of working experience. Participants in this study were 63 people, including managers (4) physicians (4), nurses and staff (44), social workers (4), security guards (4), and patients carriers (3).

An interview guide that was designed according to the objectives of the research was used to conduct interviews. The interview questions (Additional file 1) focused on identifying the reasons for patient absconding. These questions were designed by reviewing related literature to patient escape, then reviewed by the research team, and its shortcomings were addressed. All interviews were conducted in the workplace of the participants. The interviews were recorded with the written consent of the participants prior to the interview, and in cases where the interviewees did not agree to record the interview, the interview was written down. Each interview lasted for 20–40 min. Then, the interviews were transcribed, coded, and initially analyzed.

To evaluate the qualitative data that leads to increased validity and generalizability of the results, measures including, spending enough time to conduct interviews, sampling with more variety and from different treatment-related groups, preparing interview questions with the exchange of research members and literature, confirmation of the interview transcripts by some of the interviewees and careful review of the coding by the research members. After the 63rd interview, it was determined that the saturation point had been achieved, i.e., no new information was emerging to answer our study questions. The reason for the greater number of interviews is the more in-depth investigation and achieving a greater number of reasons in connection with the research topic.

### Analysis

Colaizzi’s content analysis (CCA) was used to analyze the interviews [16]: At first, all interviews were transcribed and reviewed several times. In the next stage, the texts were read several times, and the important points were underlined to be distinguished from other parts. Then, the important sections were broken into the smallest meaningful units. In the following stage, the less related or irrelevant data were eliminated. The themes were placed in some groups according to their frequency and meaning, where name of the groups indicated the content of the group and the purpose of the participants. The groups were reviewed several times, and similar groups were combined. Finally, the researchers agreed upon the meaning of the data and what appeared as the themes and subthemes, contents, and their names.

The qualitative analyses followed Lincoln and Guba’s quality criteria for ensuring trustworthiness, which were credibility, transferability, dependability, and confirmability.

To ensure the data’s credibility, extensive time was spent on data collection and analysis, and the interview questions were reviewed by research members and based on valid articles in terms of content. To ensure transferability, purposeful sampling was used, and the interview was conducted with knowledgeable and well-informed interviewees. To ensure the dependability criteria, all details of the interviews were carefully recorded and coded, with discussion and argument between the research team, and then the team by organizing, reducing the volume of data, and evaluating and controlling the results of combinations, the final codes were extracted. Confirmability is provided by interviews and documentation of the research process.

No special software was used to carry out these stages, and all stages were carried out manually. The initial framework included 3 Themes concerning the reasons for patient absconding. However, the subgroups of each of these components underwent fundamental changes. In the findings section, the letter “P” along with a number indicates the participant who has been quoted.

## Results

According to the findings, the majority of the 63 participants in this study were women (48 people - 68.2%). The majority of interviewees were married with more than 5 years of work experience. Following the analysis of the qualitative findings, the interviewees identified and classified the sub-themes from the mentioned topics in order to identify the challenges of patient absconding from hospitals. In the present study, 3 Themes and 15 subthemes were identified, explaining the reasons relating to patient absconding behavior (Table 1).

### First category: economic factors

#### Lack of insurance coverage

Universal health insurance coverage means all citizens can access and afford proper services, including preventive, therapeutic, and rehabilitation services, when needed. This issue is helpful in the realization of the goals of the health system. Accordingly, one of the main aims of the Ministry of Health and Medical Education (MOHME) is health coverage and an increase in the population of base health insurance. This objective has been sought in the health system reform plan. Despite the presence of these governmental plans and policies, insurance coverage has not yet been implemented thoroughly. In this study, many interviewees posited insurance noncoverage as one of the challenges of patients escape from the understudy public hospitals. “*Some patients had no insurance coverage*” (P. 41).

On the other hand, some interviewees mentioned a lack of insurance coverage as well as a budget for the insurance of some patients like cardboard sleepers whose reception and settlement of therapeutic costs have brought about difficulties to hospitals. In this respect, one of the participants believed that: “*some people, like cardboard sleepers, come to hospitals with false identities, and they aim to escape from the beginning so that their identities are not disclosed. These people comprise many numbers of escaping individuals. These individuals have no identities and are re-received by the hospital emergency. There is no budget for the insurance of these patients so that some part of the cost these patients undergo for hospital payment can be compensated by insurance coverage*” (P. 63).

#### Lack of proper and complete insurance coverage

Despite the availability of health insurance coverage, patients cannot afford therapeutic expenses due to insufficient support and improper coverage, and they escape from the hospital without settling. Concerning this issue, participants said that: “*some patients needed urgent and emergency cares, such as suicide and quarrel cases. They had to pay the costs themselves since their costs were not covered by insurance. Thus, they escaped from the hospital without settling, and, hence, making hospitals face difficulties. For accident patients with high costs and without any supportive insurance coverage, there is an extreme tendency for escape*” (P. 42). *The patients who had quarreled escaped when their statuses become stable since insurance does not cover these cases*” (P. 40).

#### Inability to pay costs

Due to economic problems and lack of proper insurance coverage, some patients cannot afford the hospital costs and escape from the hospital after receiving therapeutic services. “*inability to pay charges has the highest effect on the escaping patient. The expenses of these patients are high and make the hospitals encounter financial problems, especially our educational hospital that is trauma center*” (P. 37). *The main reason for the escape of patients is their unaffordability*” (P. 1).

#### Receiving foreigners

The reception of foreigners in some hospitals leads to many problems owing to their high care costs and insurance noncoverage, and, in some cases, it results in their escape without settling. In this regard, the participants told that: “*The majority of our runaway patients are foreigners whose therapeutic costs are high, and they cannot afford the payments*” (P. 46). “*Illegal foreigners with no identification cards are not covered by any insurance, and thus they have a high tendency to escape. Similarly, the runaway statistics of this community is high*” (P. 42).

### Second category: patients’ social and behavioral factors

#### Addiction

Concerning the geographical conditions and situations of the understudy hospitals, one of the social problems they face is the patients’ addiction. Many of these individuals belong to low social class, and they cannot tolerate the hospital conditions when they are hospitalized and treated. They run away in some cases before their care processes are completed. “*Some escaping patients are addicts and have social and familial problems*” (P. 26). “*The hospitalized addicts cannot tolerate the hospital at their hangover time, and they are compelled to escape*.” (P. 1).

#### Suicide and illegitimate pregnancies

Illegitimate pregnancies and suicide are two social intricacies leading to the runaway of the patients more than the other factors: “*The reason for the patients’ runaway is poisoning. Those patients that commit suicide and are transferable to psychological hospitals escape mainly due to social problems and difficulties*” (P. 62). “*Our main intricacy is illegitimate pregnancies. Patients leave their babies and run away. It gives rise to many legal and civil as well as cost problems for hospitals, and we spend a lot of time to pursue these individuals*” (P. 63).

#### The free hospital service misconception

The patients of the studied hospitals were often from deprived areas or enjoyed lower social and economic statuses. They faced catastrophic care costs due to their lack of insurance or inappropriate insurance-service coverage, and they were worried about their received therapeutic service charges. After receiving care services, some individuals with false identities aimed to run away from the hospital without paying the costs. “*Some think that hospital services are free; they come with false identities and aim to escape from the beginning so that their identities are not illuminated. These individuals comprise the main body of patients escaping from the hospital after receiving services*.” (P.63). *Some patients also refer to public hospitals to merely receive several paraclinical services since they are less costly than private hospitals, and they escape after they receive paraclinical services from the hospital. “Some patients come with a prior motivation, fulfill their paraclinical demands, and escape the hospital*” (P. 42).

#### Nonsupportive families

Disease impresses both the patient and his family. Anxiety, stress, and inconvenience derived from a person’s illness are transferred to his family as well. For patients, a family is a defensive shield against problems; however, some families are incapable of supporting their patients owing to diverse reasons, such as economic and familial problems, such that some families were unaware of their patients in this study. *Some escaping patients need their families’ approval for their discharge due to legal impediments. It is because they may aim to commit suicide, and they do not want their families to be informed*” (P. 1). *Some also run away due to familial problems. Perhaps, some cases of these individuals also had familial problems, and their families were unaware of their problems*” (P. 2). “*Many of these patients have no families*” (P. 35).

In contrast, some cases of the patient escape, such as the runaway of infants, children, and adolescents are fulfilled by the support of their families, friends, or carers. “*Friends and entourage can influence patients’ runaway*” (P. 61). *In some cases, it is families that scare away the patients, particularly infants and children* (P. 61).

#### Mental and psychological problems of patients

The escape of a patient is a deflective behavior resultant from varying factors that might be associated with the behavioral and mental problems of the patient. These problems may have roots in disease diagnosis and treatment process, as well as dissatisfaction with hospitalization in the hospital. The research participants referred to such social and behavioral problems of the escaping patients and told that: “*Many patients have mental and psychological problems and do not stand the treatment process*” (I. 48).

On the other hand, some interviewees mentioned some different factors of the patients’ behavior for running away and declared that: “*The reason for the escape of these individuals is that they think they have been cured, and they do not need to attend the hospital and continue their treatment. Some people are wicked, and they do not like to be in the hospital milieu and observe the laws and regulations*” (P. 59). “*The escape of the patient may be due to his disease diagnosis. The escaped individual may be among those who do not want to be hospitalized. These issues reflect the mental and familial problems of the patient. Those patients that are forced to be hospitalized run away more probably* (P. 61).

### Third category: hospital-related factors

#### Horizontal hospitals building structure and insufficient supervision

Due to the provision of a wide number of services and activities, hospitals enjoy numerous physical conditions. The presence of diverse and crowded wards, as well as numerous doors in the hospital environment, minimizes the possibility of control and supervision, and this issue paves the way for the escape of some patients from the hospital. In this respect, one of the participants believed that: “*The dispersion of the wards and the availability of numerous doors can be the reason for the runaway of the patient*” (P. 1).

In hospitals, the guardians and police forces have crucial roles in the establishment of order and observance of regulations. Owing to its environmental conditions and outnumbered clients, controlling and supervising hospitals is difficult, making these forces confront problems in the execution of their responsibilities. Likewise, the negligence and slumber of the guardians can sometimes trigger reduced control and supervision, as well. In this regard, some participants believed that: “*The negligence of nurses and disregard of the guard forces can result in the escape of patients*” (P. 51). “*The entrance and exit of individuals to and from hospitals are not controlled, and they think that they can easily run away*” (P. 26). Moreover, some other participants referred to the few numbers of guardians in wards and thus decreased control and supervision in hospitals and told that: “*The shortage of the guard forces in hospitals is one of the main reasons for individuals’ escape. If they are more in number, more protection and care will be realized*” (P. 8). “*The number of guard forces in hospitals is few, and they do not properly accomplish their responsibilities*” (P. 35).

#### Lack of training and informing patients

In health-promoting hospitals, training and informing patients are introduced as one of the conditions and infrastructures of health promotion. This training and informing in hospitals can involve some cases such as training about the disease and its treatment process and informing about therapeutic costs, disease management, and lifestyle development in such a way that this awareness enhances the post-discharge health. Therefore, one of the standards of the health-promoting hospitals associates with patient’s interventions and information and refers to training and informing patients and their families. Unfortunately, this issue is not well executed in the majority of hospitals. In this respect, according to the prospects of the many participants, one of the factors of patient escape is the lack of patient and carer training and informing. “*Not informing and training patients is the reason for their escape* (P. 27). “*It is the unawareness and culture of the patient who should be involved and in contact with the hospital for the continuation of his therapy*.” (P. 2).

On the other hand, some participants perceived the non-training and non-informing of patients from the official processes of the hospitals and therapeutic costs as the reasons for their escape: “*Many individuals run away due to being unaware of the expenses as well as the official and discharge processes*.” (P. 32). “*It is due to patients’ unawareness of costs*.” (P. 31). “*They escape mostly owing to not being informed about costs*.” (P. 53). “*Individuals are unaware. If we inform the patient and his carers, it will decrease the dissatisfaction and runaway of the patient. Some part of it relates to the nonpayment of the costs; however, it can be solved by social-aid counseling, informing, and explaining the cost discounts.*” (P. 28).

#### Absence of a certain process pursuing patient escape

Concerning the opinions of the participants, there is no specific process in hospitals for pursuing the patient escape. Thus, no helpful coordination, planning, and practice are carried out for the minimization of this intricacy. In this regard, some participants expressed that: “*There should be a law, which makes it easy how to behave and pursue these patients*” (P. 46). “*There is no proper process for the pursuance of escape, and no guardian and ward takes responsibility. Furthermore, there is a lack of cooperation and coordination between guardians and nurses concerning the patient’s escape*” (P. 62).

#### Patients’ dissatisfaction with hospitals

The problems of patient satisfaction and the observance of his rights are significant. They are the results of a wide group of varied tasks that should be prioritized by the hospital management. The healthcare employees and personnel of hospitals should attempt to attract patients’ satisfaction with services. The dissatisfaction of patients gives rise to some problems like noncooperation with the healthcare personnel as well as probable complaints and skirmishes, and sometimes patients leave the hospital or run away. The interviewees referred to some dissatisfaction-related problems, which can have parts in the escape of patients from the understudy hospitals. In this regard, they noted that: “*A patient may be dissatisfied with the current practices and conditions of the hospital*.” (P. 15). “*Dissatisfaction with the hospital personnel and their cares, dissatisfaction with the facilities present in the hospital, and bad treatments with patients play roles in the escape of patients*” (P. 34). “*Lack of effective communication of the healthcare personnel with the patient can be a reason for the patient’s escape*.” (P. 53). “*The reason for escape stems from the incoordination of extra-organizational institutions*.” (P. 49).

Some patients expect further care from the hospital personnel, and the non-realization of this expectation triggers dissatisfaction, and sometimes the abandonment of the hospital by the patient. Some interviewees also posed: “*Perhaps, another factor for dissatisfaction is the treatment process. The patient may think that the personnel have not embarked on his treatment, and thus he gets into trouble with the personnel and physician*” (P. 27). “*No proper therapeutic action is performed, and the nurse does not stand beside the patient’s bed*” (P. 63).

#### Difficulties in the discharge process of the patient

The discharge process starts from the time the patient is allowed to be discharged to the time he leaves the hospital. This is one of the main processes of the hospital and also a fundamental challenge in hospital management. The long discharge time leads to reduced service quality as well as patients’ dissatisfaction, and, in some cases, patients leave the hospital without finishing the phases of this process and receiving the discharge sheet. In this regard, some participants believed that: “*The discharge bureaucracy (we cannot do anything about it) triggers to the runaway of individuals who do not have the patience for official processes as well as official reception and discharge tasks*” (P. 1 & 12). *The long process of the service provision and discharge leads to the impatience and escape of patients*” (P. 46).

## Discussion

In this study, the patient escape was linked to economic, social, behavioral, and hospital-related factors. The present study showed that the insurance problems of escaping patients mostly associate with cardboard sleepers, quarreling and skirmishing people, and foreigners. This problem was more evident in poor patients, cardboard sleepers, and the other deprived community of the society, and hospitals do not tend to render services to these patients since many of the cardboard patients escape from the hospital without paying the costs and insurance coverage, and even after receiving healthcare services several times. Thus, they can impose extensive costs on hospitals.

According to some studies, the rate of patients absconding from psychiatric hospitals is higher than in general hospitals, and the majority of runaway patients are young, male, have mental disorders, and have a history of escaping from the hospital [13, 17–20].

According to the findings of the current study, one of the crucial factors in patient escape is individuals’ inability to pay costs. Zarei et al. also mentioned families’ unaffordability as one of the reasons for patient escape [14]. Concerning the low economic, social, and cultural levels of the understudy society and fair inaccessibility to health services, some steps should be taken so that all people in the society fairly access healthcare services, and we do not witness any escape due to unaffordability or lack of insurance coverage. Thus, the policymakers can realize fairness in using healthcare services by providing insurance coverage, which covers all hospitalization and outpatient services. They can also support low-income and poor groups, elderlies, and disabled ones by rendering payment exemptions. The results of the mentioned studies are in line with the prospects of the participants in the present research.

Concerning the ascending trend of emigration of foreigners, especially Afghans, the need for attention to the health services of this population becomes more evident. The outnumbered presence of these foreigners in the south-east of the country gives rise to the further reception of these patients in the healthcare centers of these areas. Due to lack of insurance coverage of these patients along with their high therapeutic costs, many of them escape from these centers, and this issue oppresses the authorities and employees of these hospitals. Hence, regarding the concerns posed in this study, the necessity for noticing these problems by the policymakers of the health system is felt more than ever. In Falkowski et al.’s study, many examined escaping patients were the Caribbean African foreigner [21].

In this study, many behavioral and social challenges of patients’ escape were related to addiction and its treatment process, poisoning, suicides, illegitimate pregnancies, patients’ mental and behavioral disorders, nonsupportive families, and individuals’ misconception regarding the freeness of hospital services. Numerous studies have referred to addiction and its treatment process as a runaway factor. In the study by Gerace et al., many escaped patients were addicts and abused drugs [22]. Ajalli et al. reported that many escaping patients abused drugs [23].

Poisoning and suicides were other reasons for the escape of patients in one of the studied hospitals. After receiving health services and during their transference to wards, these patients escaped from the hospital owing to the ward crowdedness, dissatisfaction with transference to a psychiatric hospital, fear of the hallmark of the mental patient, and absence of separate wards or rooms for mental patients in the wards of the hospital. The majority of the interviewees posited that another reason for the runaway of these patients is the nonsupport of their families and the few visits of families from patients. In this regard, Mosel et al. referred to patients’ loneliness, inaccessibility, and non-contact with their families as the reasons for their escape [19]. To increase familial supports from patients and decrease their escape due to social and familial problems, we can design and implement psychological interventions such as determining social-psychological needs, providing mental education programs to family members, employing anxiety-reducing techniques, and rendering therapeutic information to family members [24]. Illegitimate pregnancies and cardboard sleepers were also the social intricacies leading to the escape of patients in the understudy hospitals, while the development of social safety is crucial and essential in decreasing these kinds of social problems. A study showed that the attributes of the escaping patients did not differ from non-escaping ones in the control groups. This implicates that a tendency to escape is not merely related to the patients’ personality and behavioral characteristics; rather, other factors, including the social context, have contributions, as well [25].

The results of this study revealed that dissatisfaction with facilities and environmental conditions of the understudy hospitals had a small effect on the patients’ escape; however, these cases of patient escape can be reduced by improvements in the conditions and equipment of hospitals, proper patient-physician/nurse relationships, and provision of information on the treatment process and expenses.

The examination of the present condition showed that not quickly deciding on the discharge of the patient, long discharge process, and unawareness of patients and their families of the discharge process in some understudy hospitals lead to their impatience and non-fulfillment of this process, and many of them leave the hospital without taking the discharge sheet and completing this process. These patients do not settle completely and maybe reckoned as an escaping patient. Difficulties in the discharge process have been addressed in many of the studies on the hospitals of Iran and lead to the dissatisfaction of patients and personnel. Furthermore, one of the subtopics posed in this study is that the provision of no information respecting the treatment cost and process brings about this misconception in some individuals that the healthcare services are free in hospitals. Thus, it impacts the escape of the patients from the understudy hospitals, although no study has so far documented this problem. The lack of a process pursuing the runaway of patients, as well as the lack of laws and executive approaches to treating the escape of patients, is of the main challenges of the field according to the viewpoints of the interviewees. Many interviewees asked for a vivid and clear process for treating the intricacy of patients’ escape from hospitals. Similar to other studies, this study enjoyed some limitations. First, it investigated the escape of patients solely in public hospitals. Thus, future studies that probe into other hospitals are needed. Second, owing to their inaccessibility, the escaped individuals were not interviewed. Therefore, we suggest future studies specifically examine these patients. Third, the limitation of qualitative research is the possibility of researcher bias. The current study tried to reduce the influence of the individual researcher’s values and understandings on data interpretation by incorporating two researchers in the development of codes, themes, and a thematic framework, both of whom worked reflexively. Also, to reduce potential bias, we used the opinions of a diverse variety of participants and continued to recruit and analyze data until data saturation was achieved across all groups of individuals.

## Conclusion

Patient absconding, as one of the most important and challenging issues in hospitals, in addition to economic consequences, can also have social consequences that were addressed in the present study. The findings of our study suggest that in order to solve the problem of patient absconding, all treatment staff related to the patient in the hospital should participate in solving it, and everyone should do their part in solving this important problem to reduce the problem of hospital escape. This study also helps to understand the importance of patient absconding better as well as prevent and reduce patient absconding from hospitals.

## Data Availability

Anonymised transcripts are available from the corresponding author.

## Abbreviations

CCA: Colaizzi’s content analysis
MOHME: Ministry of Health and Medical Education

## Footnotes

1 - Official currency of Islamic Republic of Iran, each US$ equaled 120,000 rials at the time of present study
